# Reforming sanitary-epidemiological service in Central and Eastern Europe and the former Soviet Union: an exploratory study

**DOI:** 10.1186/1471-2458-10-440

**Published:** 2010-07-27

**Authors:** George Gotsadze, Ivdity Chikovani, Ketevan Goguadze, Dina Balabanova, Martin McKee

**Affiliations:** 1 Curatio International Foundation, 37 d Chavchavadze ave, Tbilisi, Georgia; 2 European Centre on Health of Societies in Transition and European Observatory on Health Systems and Policies, London School of Hygiene and Tropical Medicine, Keppel Street, London WC1E 7HT, UK

## Abstract

**Background:**

Public health services in the Soviet Union and its satellite states in Central and Eastern Europe were delivered through centrally planned and managed networks of sanitary-epidemiological (san-epid) facilities. Many countries sought to reform this service following the political transition in the 1990s. In this paper we describe the major themes within these reforms.

**Methods:**

A review of literature was conducted. A conceptual framework was developed to guide the review, which focused on the two traditional core public health functions of the san-epid system: communicable disease surveillance, prevention and control and environmental health. The review included twenty-two former communist countries in the former Soviet Union (fSU) and in Central and Eastern Europe (CEE).

**Results:**

The countries studied fall into two broad groups. Reforms were more extensive in the CEE countries than in the fSU. The CEE countries have moved away from the former centrally managed san-epid system, adopting a variety of models of decentralization. The reformed systems remain mainly funded centrally level, but in some countries there are contributions by local government. In almost all countries, epidemiological surveillance and environmental monitoring remained together under a single organizational umbrella but in a few responsibilities for environmental health have been divided among different ministries.

**Conclusions:**

Progress in reform of public health services has varied considerably. There is considerable scope to learn from the differing experiences but also a need for rigorous evaluation of how public health functions are provided.

## Background

The instigators of the 1917 Russian revolution initially placed a high priority on population health, for sound political reasons; ten million people succumbed to the epidemics of typhus that afflicted Russia from 1918 onwards, threatening the survival of the new regime. The first People's Commissar for Public Health, Nikolai Semashko, who gave his name to the Soviet health care system, created an extensive public health infrastructure, based on a network of sanitary-epidemiological (san-epid) stations, owned by the Ministry of Health, and charged with surveillance and control of threats to health. These threats were interpreted mainly as infectious diseases and control of some forms of environmental pollution (although in both cases always giving priority to the needs of the state) [1]. In the post-war period this system was extended into the three Baltic States, by now absorbed into the USSR, as well as the Soviet satellite states in central and Eastern Europe (CEE). Initially, a great deal was achieved, especially in relation to control of vector-borne and vaccine preventable disease. However, the system was unable to respond to the evolving challenge of non-communicable diseases, whose risk factor lay in the choices made by individuals, shaped and constrained by their environments [2].

The dramatic social, political and economic changes that followed the fall of the Berlin Wall in 1989 highlighted the failings of the existing system. Some of the newly independent states that had emerged from the USSR experienced outbreaks of vaccine-preventable disease such as diphtheria and the re-emergence of malaria which the USSR had previously eradicated. Traditional authoritarian and medicalized approaches to disease control offered no answers to the new threat from HIV, concentrated initially in a marginalized population of drug users [3-5]. Nor could they respond to the threats posed by ubiquitous cheap alcohol and the aggressive marketing by transnational tobacco companies [6,7]. The centrally planned and managed san-epid system was, in many countries, increasingly misaligned with new models of governance, in which the role of the state was changing rapidly, with power often being decentralized, in some cases to new actors in the private sector [8]. The result was that certain areas of direct public health relevance, such HIV/AIDS, sexually transmitted infections and substance abuse were now run through vertically organized programs with separate systems of financing and management and poor linkages with other services [5,9].

Non-governmental and voluntary organizations often moved in to fill gaps in public health provision, often benefiting from technical and financial support from western donors. This was especially so in relation to vulnerable populations that would otherwise be neglected, such as measures to reduce the risk of HIV transmission among injecting drug users and commercial sex workers in some parts of the former Soviet Union. Some of these organizations now play a major public health role [10].

Two decades on, the public health systems in the former communist countries have had to change, with in many cases the direction of change influenced by the availability of external donor funds. Yet many of these changes have taken place away from public gaze, attracting little attention from researchers and policy analysts, whose focus has more often been on topics such as equitable financing and effective delivery of health care.

In this paper we seek to redress this balance by reviewing the reforms that have taken place in these countries, focusing on how the former san-epid systems have responded to pressure for decentralization, diversification of funding sources, and allocation of tasks in a new, more pluralist institutional environment. The paper is descriptive; evaluations of the new structures are clearly needed but are beyond the scope of this paper.

## Methods

A review of relevant literature was conducted to answer the question "how, if at all, have san-epid systems changed?" The review included all those that had some form of san-epid system in place (the basic model was the same everywhere but there were some national variations); this included the twelve countries that had been part of the USSR since its establishment (further referred as former Soviet Union (fSU)), the nine that had been occupied by the USSR after World War II (including the Baltic States and the Czech Republic and Slovakia having separated in 1993), and Albania. The public health system was quite different in Yugoslavia so the countries emerging after its breakup were not included in the analyses.

The search strategy was iterative, beginning with the names of individual countries plus " "Public Health System" "Public Health Functions", "Health Care reform", "Public Health financing", "Public Health Reform", "Public Health Services", 'Public health decentralization", "Health Sector Reform", "Ministerstvo Zdravookhranenia", "Prikaz", "Ukaz", "Sanitarno-Epidemiologicheski Nadzor", "San-Epid Services", "Public health Law", "Local Governance Law". Searches were made on PubMed and using Google, as well as dedicated searches of web sites of ministries of health, relevant government agencies, and international organizations and donor agencies, including the World Health Organization, World Bank, UNICEF and European Observatory on Health Systems and Policies. References identified on initial searches were followed up. National Health Accounts were examined to track funding flows. Searches were conducted in English, Russian and Georgian and covered the period 1990-2009.

### Conceptual Framework

**Figure 1:**
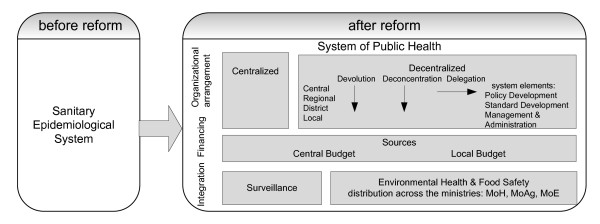
**Conceptual Framework**.

Following extensive discussions with experts in public health, and drawing on the experience of the authors, a conceptual framework was developed to guide the review (Figure 1). In this framework the term "*Sanitary Epidemiological System*" designates the public health services in each country prior to the changes in 1989-91. The term "*system of public health" *denotes the system that is necessary to implement essential public health functions effectively. We focus on the two main functions undertaken by the san-epid system, *communicable disease surveillance, prevention and control *and *environmental health*. Modern public health goes far beyond these, for example as set out by the World Health Organization in a list of *Essential Public Health Functions*, but to the extent that these functions are undertaken at all in this region, they have largely fallen within the remit of new entities [11].

Decentralization was a dominant trend in public sector and health sector reforms in this region during the 1990s. It was seen explicitly as a departure from the existing centralized planning system and was envisaged as bringing improved efficiency, cost-consciousness, enhanced accountability, and decisions tailored to local needs. Consequently, we paid particular attention to the process of decentralization as it affected the public health system. We employ the classification of decentralization proposed by Rondinelli (1983), with its four types [12]:

a. *Devolution *(political decentralization) i.e. devolving responsibilities to lower level political authorities such as regional or municipal governments.

b. *De-concentration *or transfer of responsibilities and power from the centre to the periphery within a formal administrative structure.

c. *Delegation *transfer of functions to more or less autonomous public organization.

d. *Privatization *- when responsibilities for a particular function is transferred from public to private actors [13].

The public health functions we were studying are everywhere in this region seen as a core role of government, although we do note that, in many countries international agencies and non-governmental organizations (NGOs) do participate actively in some public health functions, especially in relation to HIV/AIDS and tuberculosis. We did not examine services for those with HIV/AIDS and tuberculosis, since these were not part of the historical san-epid system and still function as autonomous but centrally directed sub-systems with their own convoluted funding sources. Furthermore, these services have been the subject of extensive research already [14-16].

Our initial analysis found that, where decentralization had occurred, it had not affected all the aspects of the work of the san-epid system to the same extent. Hence, we examined separately four elements of the system such as: a) *policy development*; b) *standard setting*; c) *management and administration*; and d) *financing*. With respect to the last category we looked at whether funding for public health activities came from central, regional or local government budgets. It was often difficult to differentiate the last two of these, although it was possible to ascertain that such funding was sub-national. If funding for public health was made through ring fenced transfers to local governments from central budget, the funding was treated as central. We were unable to find any evidence of private and/or donor funding of operational, as opposed to capital costs.

Finally, we examined whether the once integrated disease surveillance and environmental health and food safety functions had been kept together and, if not, where they had gone to.

## Results

The findings are summarized in four tables. Table 1 shows the current distribution of the four elements of system of public health across levels of government (central = C, regional = R and local = L) in each country. Table 2 reports distribution of the system elements among the different entities involved in delivering disease surveillance and environmental health functions in the Central and Eastern European countries. Table 3 examines in more detail the situations in those countries that have decentralized some responsibilities. These countries are further unpacked by looking at type of decentralization using the classification proposed by Rondinelli [12]. Table 4 looks only at those countries where environmental health and food protection functions were shifted to other ministries.

**Table 1:** Distribution of public health system elements by levels of government in CEE & fSU countries

		system	elements	
	
Country	Financing	Policy development	Standards setting	Management & administration
**Central and Eastern Europe**				
Albania [29]	C	C	C	C
Bulgaria [30,31]	C	C	C	C, R
Czech Republic [17,32]	C	C	C	C, R
Estonia [25,33,34]	C; L	C	C	C, R, L
Hungary [35]	C	C	C	C, R
Latvia [24]	C	C	C	C; R
Lithuania [36-38]	C	C	C	C; R
Poland [39]	C, L	C	C	C, R
Romania [18,40]	C	C	C	C, R
Slovakia [20,41]	C	C	C	C, R
**Former Soviet Union**				
Armenia [42]	C	C	C	C
Azerbaijan [43]	C	C	C	C
Belarus [44,45]	C	C	C	C
Georgia [22,46,47]	C, L	C	C	C, R, L
Kazakhstan [48,49]	C	C	C	C
Kyrgyzstan [50]	C	C	C	C
Moldova [51,52]	C	C	C	C
Russia [53,54]	C	C	C	C
Tajikistan [55,56]	C	C	C	C
Turkmenistan [57]	C	C	C	C
Ukraine [23,58]	C	C	C	C
Uzbekistan [59-61]	C	C	C	C

C = Central; R = Regional; L = Local

**Table 2:** Administrative tiers, public health entities and system elements in Central and Eastern European countries

country	administrative tiers	institutions		system	elements	
			
			financing	policy development	standard setting	management & administration
Albania [29]	Central	Ministry of Health (Public Health Department; Chief Sanitary Inspector)	x	x		x
		Institute of Public Health^a^			x	
	Regional (prefectures)	n/a				
	District	District Public Health Directorates^b^				x
	Local (municipality)	n/a				
Bulgaria [30,31]	Central	Ministry of Health (Principal State Health Inspector)^c^	x	x		x
		National Centre for Public Health Protection^d^			x	
		National Centre for Infectious and Parasitic Diseases				
	Regional	Regional Inspectorate of Public Health Protection and Inspection (RIPHPI)				x
	Local (municipality)	n/a				
Czech Republic [17,32]	Central	Ministry of Health (Chief Public Health Officer; Department of Hygiene, Epidemiology and Microbiology)	x	x		x
		National Institute of Public Health^e^			x	
	Regional	Regional Public Health Authorities^f^ Institutes of Public Health^g^				x
	Local (municipality/community)	n/a				
Estonia [25,33,34]	Central	Ministry of Social Affairs (Public Health Department)	x	x	x	x
		Health Protection Inspectorate (HPI)				
	Regional	County office of the HPI				x
	Local	Municipal offices^h^	x			x
Latvia [24]	Central	Ministry of Health (Public Health Department)	x	x	x	x
		Public Health Agency^i^				
		State Sanitary Inspectorate				
	Regional	Public Health Center^j^				x
Lithuania [36-38]	Central	Ministry of Health	x	x	x	x
		State Public Health Service				
		Center for Communicable Disease Prevention and Control (CCDPC)^k^				
	Regional (Apskritys)	County Public Health Center				x
	Local (municipalities)	Local branches of County Public Health Centers				
Hungary [35]	Central	Ministry of Health, Social and Family Affairs	x	X		
		National Public Health and Medical Officer Service (NPHMOS)^l^			x	x
	Regional^m^	n/a				
	County	County Office				x
	Local (municipality)	Municipal Office				
Poland [39]	Central	Ministry of Health General Sanitary Inspectorate (Chief Sanitary Inspector)	x	x	x	x
		National Institute of Hygiene^n^				
	Regional (voivodship)	Sanitary Inspection station	x			x
	District (powiat, county)	n/a	x			
	Local (gmina, commune)	n/a	x			
Romania [18,40]	Central	Ministry of Public Health (Department of Public Health)	x			x
		Public Health Institutes at Universities^o^ National Center for Disease Control^p^ National Institute for Research and Development in Microbiology and Immunology "Cantacuzino"^q^		x		
	District (judet)	District Public Health Authorities (DPHA)				x
Slovakia [41]	Central	Ministry of Health	x	x		x
		National Office of Public Health			x	
	Regional	Regional Offices of Public Health^r^				x
	Local (municipal)	n/a				

^a ^The Institute of Public Health (IPH) is a national body, responsible to the Ministry of Health.

^b ^De-concentrated facilities of the Institute of Public Health

^c ^The Principal State Health Inspector within the Ministry of Health provides technical oversight of public health services within both the heath care sector and other ministries such as defense, interior, transport and justice. The public health network is centrally managed and consists of 28 RIPHPIs and some specialist national facilities.

^d ^Part of the Public Health Network, providing methodological support for health protection and disease control, environmental health, occupational health, and food and nutrition policy for regional inspectorates, regulatory bodies, and laboratories.

^e ^The National Institute of Public Health undertakes research, provides technical advice on hygiene, epidemiology, microbiology, occupational health, environmental health, and health promotion; drafts proposed legislation, and participates in under- and post-graduate training.

^f ^Regional Public Health Authorities are responsible for epidemiological surveillance, logistical support for immunization programmes, and health inspections.

^g ^Regional Public Health Institutes undertake laboratory analyses, including support for Regional Public Health Authorities, evaluate living and working conditions and quality of consumer and industrial products.

^h ^Municipalities are responsible to monitoring compliance with health protection legislation and coordinating disease preventive programmes.

^i ^The Public Health Agency was created from the National Environmental Health Centre (which was the redesignated Sanitary Epidemiological System from the Soviet period)

^j ^Public Health Centers are the regional offices of the Public Health Agency

^k ^The CCDPC is responsible for epidemiological surveillance of communicable diseases (with the exception of AIDS, HIV, STDs and tuberculosis).

^l ^The NPHMOS is under the auspices of the Office of the National Chief Medical Officer and is supported by two national bodies: the Fodor József National Centre of Public Hygiene, with five national institutes covering occupational health, chemical safety, nutritional health, radiation safety and environmental health; and the Johan Béla National Centre of Epidemiology.

M Regions are groupings of counties. Their creation has been controversial and they currently have only limited functions. This may, however, change.

^n ^The National Institute of Hygiene oversees scientific monitoring of biological, chemical and physical hazards to health, as well as undertaking research and training in technical and scientific aspects of surveillance.

^O ^Four institutes in Bucharest, Cluj, Iasi, and Timisoara are autonomous bodies accountable to the Ministry of Health, providing technical support on public health and related topics to ministries and other national institutions with health responsibilities. The Bucharest Public Health Institute elaborates national standards and regulations, provides technical coordination, and implements four programmes within the National Programme on Community Health.

^p ^Since 2005 National Center for Disease Control has operated within the Institute of Public Health in Bucharest. Its task is to integrate parallel surveillance systems and coordinate the national communicable diseases network, monitor immunization, coordinate a national system of early warning and rapid response, and manage a public health information system.

^Q ^Along with teaching and research activities this provides technical support to public health authorities and conducts epidemiological surveillance.

^r ^The Regional Offices are the successors of the State Health Institutes, dating from 2004. They have divisions of hygiene, epidemiology and occupational medicine and are responsible for health promotion and health protection, disease prevention and monitoring of environmental impacts on health.

**Table 3:** Type of decentralization in the system of public health in CEE/fSU countries

Country	Type of decentralization		
	
	Devolution	De-concentration	Delegation
**CEE**			
Albania		M&A	S, M&A
Bulgaria		M&A	S, M&A
Czech Republic		M&A	S, M&A
Estonia	F, M&A	M&A	M&A
Latvia		M&A	M&A
Lithuania		M&A	M&A
Hungary		M&A	S, M&A
Poland	F	M&A	M&A
Romania		M&A	M&A
Slovakia		M&A	M&A
**fSU**			
Georgia	F, M&A	M&A	F, M&A

F = Financing; S = Standard setting; M&A = Management and Administration

**Table 4:** Distribution of responsibility for epidemiological surveillance in selected countries

Country	Environmental health and food safety		
	
	Food quality	Water quality	Air quality
Albania [29]	MoH and MoAg	MoH	MoH
Romania [18]	MoH	MoH and MoE	MoH and MoE
Estonia [25]	MoSA and MoE	MoSA and MoE	MoSA and MoE
Georgia [62]	MoAg	MoAg	MoE
Latvia [24]	MoH and MoAg	MoH	MoH

MoH - Ministry of Health; MoAg = Ministry of Agriculture; MoE - Ministry of Environment; MoSA - Ministry of Social Affairs

### Organizational arrangement of the Public Health System

The reforms to san-epid systems are quite diverse but the evidence available precludes a full comparative analysis. Nevertheless, certain patterns emerge. Table 1 shows a marked difference between the Central and Eastern European (CEE) countries and those of the former Soviet Union (fSU). The CEE countries have moved away from the former centrally managed san-epid system, undergoing a process of decentralization. However, the form of decentralization used and the actual level of autonomy achieved at sub-national level vary.

One development common to almost all the CEE countries is the delegation of powers to new national agencies outside the health ministry (delegation) (Table 2). This is similar to what has happened in other parts of the health system, such as the creation of semi-independent national insurance funds. These bodies typically operate with some degree of autonomy and engage in standard setting and management of certain public health functions. This process reflects a widespread process of political decentralization, often with the formal goal of increasing accountability and responsiveness to needs. In contrast, among the fSU countries, only Georgia has followed this path; other fSU countries have retained the san-epid system without major organizational or structural changes.

In general, the agencies with delegated powers have undergone a further process of de-concentration, with establishment of sub-national tiers. These are typically at regional level, although in Albania management and administrative functions have been de-concentrated to local level. Typically, there has been at least a partial transfer of decision-making powers, enforcement of regulations, and planning and managing public health activities to sub-national tiers.

In some cases, this process has also involved the separation of responsibilities. For example, in the Czech Republic, the legislative changes enforced in 2003 redefined the rights and duties of various actors in the public health arena, dividing the responsibilities of the former Hygiene Service (san-epid) between two new group of institutions: *Regional Public Health Authorities *became responsible for epidemiological surveillance (including infectious diseases) and immunization logistics (such as vaccine supply) and the functions related to laboratory investigation and monitoring of environmental hazards were transferred to the *Regional Institutes of Public Health *[17].

Devolution to elected lower tiers of government was rare and, where it happened, it was limited. Thus, in 2002, the Romanian government *de jure *devolved the public health system to 41 districts (judets) and the city of Bucharest. In theory, the judet authorities should have considerable influence on the public health function. However, as with other devolved responsibilities, the relationship between the central government and the judets has been turbulent and real power has been retained at the centre [18,19]. Thus, while *Public Health Authorities *have been established in each judet, their directors are appointed by the minister of health, subject to the agreement of the district prefect (the local representative of the Ministry of Public Administration). In Poland, reforms during the 1990s had also devolved some responsibility to the sub-national tier (the voivodship) but, since 1999, powers have progressively been transferred back to the centrally managed public health system.

There have been a number of cases of recentralization after initial decentralization, usually linked to broader reform of the public sector. For example, in Slovakia, in 2004 *State Health Institutes *that had been established in each district were closed and their functions were transferred to 36 regional public health offices [20].

### Financing

It was difficult to disentangle the funding streams in each country from either the documents available or National Health Accounts. The limited evidence permits only a very superficial assessment, and it is also known that, in some countries, facilities with laboratories derive additional income from commercial activities, although the legal status of these activities is often unclear. The central budget is the only source of official funding in almost all the fSU countries (except Georgia). Among the CEE countries the main official source of funding is from central government, although through diverse institutional arrangements. Thus, in Romania, where the *District Public Health Authorities *are, in theory, at least partially accountable to the judet administration, all expenses (including salaries, operating costs, materials, medicines, etc) are provided by the *Ministry of Public Health *[18].

In some, such as Estonia and Poland, there is co-funding from local government. The reforms to Polish public administration introduced in 1999 gave responsibility to the Gmina, or lowest tier of local governments, for funding some public health functions (preventive health and health promotion). In 2000 their main activity was action against alcohol-dependence (52 percent of the local budget). However, their role has diminished as power has been taken back by the central government. For example, since 2001, local government establishments no longer have routine access to information on sanitary conditions [21].

A radical process of devolution took place in the late 1990s in Georgia as responsibility for major public health functions was shifted to local municipalities, with central oversight. However, the budget available to the municipalities was insufficient and marked geographical inequalities emerged. This led to a decision to reassert central control. Since 2007, important public health responsibilities have been designated as *"delegated obligations*" of municipalities, paid from earmarked transfers from the state budget. In this arrangement, local public health institutions are still owned and managed by the municipal governments, which finance limited environmental health activities, while major public health functions such as surveillance are paid for by the central government. A similar process took place in Ukraine:

In 1997 the law "On Local Self-Government in Ukraine" devolved significant budgetary authority to oblast and district councils along with responsibility for much of public health. However, local public health organizations faced a division of accountability, to the Ministry of Health for compliance with norms and standards, and to local government, for funding and management. The process of decentralization led to increasing inequalities between wealthy and poor areas. In some regions, where there was a lack of sustainable sources of income, the health system became a heavy burden on local budgets. This changed in 2001, when financing was recentralized under the Ministry of Health, separating service from local authorities and ensuring that operating budgets are not influenced by local politics [22].

The Budget Code adopted in 2001, explicitly defines the types of health services and the type facilities that can be funded various administrative level budgets. Public health services and facilities may not be financed from more than one budget source and therefore the central budget is defined as a funding source for sanitary and epidemiological surveillance (sanitary and epidemiological stations, disinfecting stations, and control interventions) [23].

### Integration of public health functions

In almost all countries epidemiological surveillance and environmental monitoring has remained combined within a single organizational umbrella, although in some cases the scope of activity has increased. For example, in Russia a new agency, the Federal Service on Surveillance on Consumers Rights Protection and Human Wellbeing (Rospotrebnadzor) in addition to core former san-epid service functions, has assumed responsibility for food safety within a much broader portfolio of consumer protection.

Epidemiological surveillance and environmental monitoring functions were separated in Latvia, Lithuania and Georgia. In Latvia a new *State Sanitary Inspectorate *has been established, with responsibility for environmental monitoring transferred from the *Public Health Agency*, which has retained responsibility for epidemiological surveillance and control In Georgia, the separation of functions was part of a wider reform of public health in the late 1990s [24].

Environmental health (especially monitoring of water and air quality) and food safety has remained the responsibility of the health ministry in almost all countries. However, in a few countries the responsibilities have been divided among ministries, with the Ministry of Health, Ministry of Agriculture and Ministry of Environment typically assuming some responsibilities (Table 4). In Estonia responsibility for supervision of water quality is shared between the Ministry of the Environment (through the Environmental Inspectorate) and the Ministry of Social Affairs (through the Health Protection Inspectorate). Since 2007, responsibility for food safety has shifted from the Ministry of Social Affairs to the Ministry of Agriculture [25]. In Latvia regulation of food production and supply and sanitary inspections are the responsibility of the Food and Veterinary Service within the Ministry of Agriculture [24]. In Georgia, the Ministry of Health does not undertake monitoring of food, water and air quality but retains responsibility for standard setting.

## Discussion and Conclusions

This study has several limitations. The conceptual framework is inevitably a simplification of the myriad of country-specific factors that impact on how services are delivered. However, it does form a useful basis for future in-depth comparative studies of these important public health functions. The acquisition of information was difficult and, in some countries, the information we could ascertain was fragmentary and, at times, conflicting so we recognize that we may not have managed to capture some recent changes. From our own experience, which includes all of the countries included, we know that descriptions in official documents may, in reality, be aspirations rather than descriptions of reality. Also, we limited our search to material in English, Russian, and Georgian. We were limited to mapping public health structures and were unable to draw any conclusions on their performance. A future study might undertake case studies of how countries have responded to common public health threats, such as pandemic influenza. Nor could we assess their linkages with the rest of the health system. There is an obvious need for research on the effectiveness of public health systems in improving population health. What evidence does exist is not encouraging. A Bulgarian study found that public health institutions often work in parallel with NGOs and fail to engage in prevention activities, even though these lie within their responsibility and are funded and staffed to do so [26]. The available evidence suggests that these institutions are often under-employed and fail to build links with the rest of the health system.

Finally, we also know that it is essential to have a detailed understanding of the context (including political, socio-cultural, and health system characteristics) in which reforms take place as this can influence the meanings attributed to words. For example, our previous work has revealed how, for example, the methods used in outbreak investigation can vary considerably and, in the Soviet system was usually limited to laboratory investigation and did not employ standard case-control studies.

Nonetheless, we believe that this study, albeit exploratory, has captured the generality of trends in reform of two key elements of public health services in transition countries, as well as providing some pointers for future research.

Our findings indicate that the countries studied fall into two broad groups. As in other areas within the health system and beyond, reforms were more extensive in the CEE countries than in the fSU. Decentralization most often took the form of delegation from health ministries to separate agencies, with the aim of improving performance and accountability, with de-concentration within those agencies. The fSU countries, with the exception of Georgia, have largely retained the traditional san-epid system. The limited extent to which the public health system has been modernized may seem surprising, given the ambitious reforms of financing and delivery models, although perhaps not entirely so given the low status of public health in many western countries.

Although it is beyond the scope of this study to ask why each country instituted the reforms it did, it is possible to speculate. The CEE countries were subject to an important external stimulus, accession to the European Union. While, formally, the competence of the European Union in public health *per se *is very limited, as set out in Article 152 of the Treaty, European law has many provisions relating to public health functions, in areas such as food safety and environmental protection [27]. Even those countries that are not yet members have adopted large parts of the *acquis communitaire *as a means of ensuring access to western European markets for their products. In contrast, there was no such external stimulus in the fSU countries. Although it is difficult to establish cause and effect in a situation where countries were undergoing wide-ranging political and institutional reforms, the clear division between the CEE and fSU countries (with the exception of Georgia) is strongly suggestive that the process of, or in some countries continued aspiration to European Union accession played a major role.

Unfortunately, it is difficult to draw any conclusions about the effectiveness of the different strategies pursued and, in particular, whether decentralization improved performance, given that it coincided with many other developments. A further problem is that, while we have been able to describe the structural arrangements, we have not been able to assess the degree of functional autonomy of the various institutions. Thus, an organization established separately from a health ministry may in reality have limited autonomy if senior staff are political appointees or if they have little stability of funding. These are all areas that would benefit from future research. The past decade has seen a marked increase in the availability of information on health systems in European countries [28]. However, much of this evidence remains focused on the funding and delivery of curative services, with much less attention devoted to public health (disease surveillance, environmental health, etc.) even though this is a key component of the struggle against emerging and existing health threats. In this study we seek to make a small initial contribution to redressing the balance. Our findings confirm that the public health function has undergone reform in many countries. Thus, especially in the CEE countries, the successor organizations to the san-epid system have given up many of their responsibilities for environmental health and food safety to other bodies. However there is considerable variation in the design and content of reforms. This variety provides many opportunities for lesson learning in an area that has been subject to remarkably little evaluation. This will require much more detailed information on what is happening than is now available.

## Competing interests

The authors declare that they have no competing interests.

## Authors' contributions

Study conception and design: GG. Acquisition of data: IC, KG. Analysis and interpretation of data: IC, KG, GG. Drafting of manuscript: GG, IC, KG. Critical revision: MM, DB. All authors read and approved the final manuscript.

## Pre-publication history

The pre-publication history for this paper can be accessed here:

http://www.biomedcentral.com/1471-2458/10/440/prepub
